# Cetacean biodiversity, spatial and temporal trends based on stranding records (1920-2016), Victoria, Australia

**DOI:** 10.1371/journal.pone.0223712

**Published:** 2019-10-10

**Authors:** Chantel Sarah Foord, Karen M. C. Rowe, Kate Robb

**Affiliations:** 1 Marine Mammal Foundation, Mentone, Victoria, Australia; 2 Sciences Department, Museums Victoria, Carlton, Victoria, Australia; 3 School of BioSciences, University of Melbourne, Parkville, Victoria, Australia; CSIRO Townsville Australian Tropical Sciences and Innovation Precinct, AUSTRALIA

## Abstract

Cetacean stranding records can provide vital information on species richness and diversity through space and time. Here we collate stranding records from Victoria, Australia and assess them for temporal, spatial and demographic trends. Between 1920 and 2016, 424 stranding events involving 907 individuals were recorded across 31 Cetacea species from seven families, including five new species records for the state. Seven of these events were mass strandings, and six mother and calf strandings were recorded. Importantly, 48% of the species recorded are recognised as data deficient on the IUCN Red List. The most commonly recorded taxa were *Tursiops* spp. (n = 146) and *Delphinus delphis* (common dolphins, n = 81), with the greatest taxonomic richness (n = 24) and highest incidence of stranding events documented within the Otways mesoscale bioregion. We found no seasonal stranding patterns anywhere in the state. While our findings improve understanding of the spatial and temporal patterns of cetacean diversity within Victoria, we suggest greater effort to collect demographic data at stranding events in order to better study state-wide patterns through time. We conclude with guidelines for minimum data collection standards for future strandings to maximise information capture from each event.

## Introduction

Effective conservation of cetacean populations requires an understanding of temporal and spatial incidence, species richness and community composition, as well as demographic and life history parameters of populations within a region [1, 2]. Comprehensive data on cetacean species is inherently difficult and expensive to collect [3, 4]; however, strandings (defined in this study as; beach-cast animals, dead or alive [5]) can provide valuable information on species presence and distribution [6, 7], species composition [3, 8], population dynamics [3, 8, 9], stranding type (e.g., single or mass), anthropogenic impacts such as ship strikes and bycatch [4], health of wild populations [10], and diet [11].

Cetacean diversity in Australia is particularly rich with 45 of the 89 extant species recorded around its coastline [12] but more than half (25 species) are classified as data deficient on the IUCN Red List [13]. Much of our understanding of cetacean diversity and distributions within Australia has come from state-based stranding networks [4, 14–18], however, there is a distinct gap in knowledge within Victoria’s waters. Victoria is part of the South-east Marine Region of Australia, identified as having between 60 and 95 percent endemism [19], yet the region is already experiencing the effects of climate change with species range shifts already documented in non-cetaceans [12, 20, 21]. Therefore, compiling all known cetacean species occurrence records to better understand their diversity and distribution in the state is urgent.

Existing stranding records within Victoria are distributed across numerous datasets, agencies and institutions. In the past, historical stranding data from the state have been used to investigate interannual trends and drivers of cetacean distribution from 1920 to 1980 [17], to infer habitat range pre 1966 [22], and in nationwide species specific studies such as Kemper [23] describing *Caperea marginata (*pygmy right whale) distribution. However, there is no up to date comprehensive database of cetacean occurrences in Victoria that can be used to infer spatial and temporal patterns of species richness and community composition.

In this study, we compile and validate all known cetacean occurrence records, including strandings and specimen records, to define distributional and demographic patterns through time. We conclude with recommendations for minimum data requirements and sampling effort for future stranding events to improve conservation management and monitoring of cetacean populations and communities within the South-east Marine Region of Australia.

## Methods

### Study area

**Fig 1:**
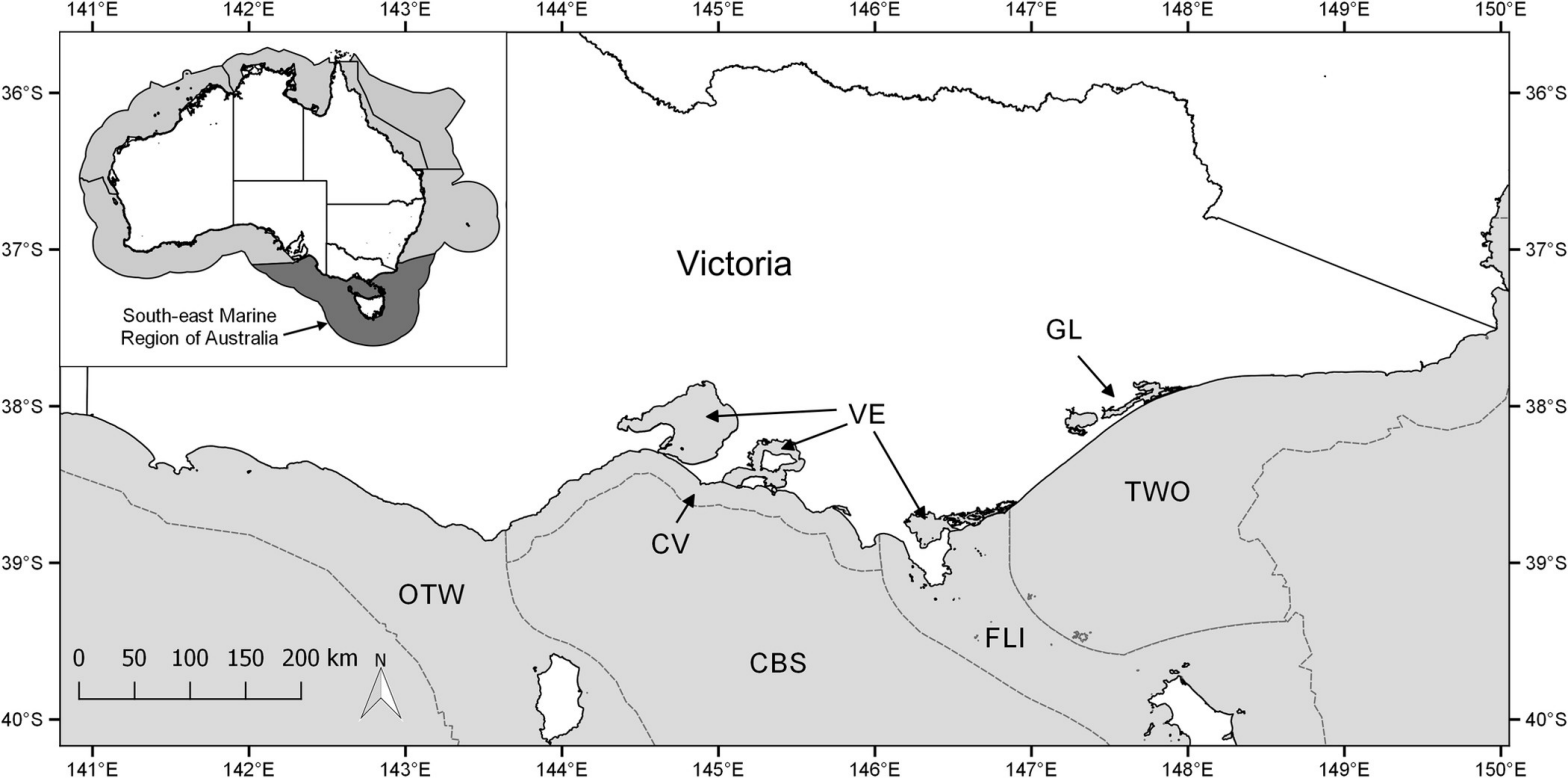
Map of Victoria, Australia with gippsland lakes (GL) and IMCRA v4.0 mesoscale bioregions [19]: Otway (OTW), Central Victoria (CV), Victorian embayment (VE), Flinders (FLI), Twofold shelf (TWO), and Central bass strait (CBS). Reproduced from Commonwealth of Australia, Australian Government Department of Environment 2016 licensed for re-use under CC BY 4.0., and Victorian State Government, Department of Environment, Land, Water and Planning, licensed for re-use under CC BY 4.0.

The South-east Marine Region of Australia stretches from southern New South Wales, to the Great Australian Bight in South Australia and across to Tasmania including the Bass Strait (Fig 1). The region is oceanographically complex, with subtropical influences from the northern East Australian Current and Subantarctic influences from the Antarctic circumpolar current, resulting in broad seasonal variation [19, 24]. Localised areas of high productivity exist during spring and autumn along the subtropical convergence zone, and seasonal upwellings are found along the Bonney Coast and Bass Cascade [24]. The region has had a relatively stable climate over a long period of time leading to a unique composition of species and high levels of endemism [19].

Victoria has 2,512 km of coastline with a narrow latitudinal extent (37°S-39°S). The coast is delineated into five mesoscale bioregions as part of the Integrated Marine and Coastal Regionalisation of Australia (IMCRA v4.0); Otway (OTW), Central Victoria (CV), Victorian embayment (VE), Flinders (FLI), and Twofold shelf (TWO) (Fig 1). The IMCRA is a spatial framework for Australia based on the ecology of the marine environment and is used to assist with regional management and planning [19].

### Taxonomy

In this study, we used taxonomy from Jackson and Groves [25]. Of note, we include *Tursiops australis* (Burrunan dolphin) in the species list. Whilst we acknowledge the validity of *T*. *australis* remains contentious [26, 27], since the initial species description [28], a larger body of genetic evidence further validates *T*. *australis* as a separate species, sister taxa to both *Tursiops aduncas* (Indo-Pacific bottlenose dolphin) and *Tursiops truncatus* (common bottlenose dolphin), using mtDNA regions [29], concatenated mtDNA/nuDNA sequences [30], the mitogenome [30–33], and more recently in the time calibrated molecular phylogeny of Certiodacyla [34]. We assigned a taxon to each record based on multiple criteria; where available we used existing genetic analysis, skull and external morphology and museum specimen metadata. For records where specimen or other detailed morphological data were lacking, we assigned the record to the nearest taxonomic rank, i.e. *Globicephala sp*. are undefined species within the *Globicephala* genus; Cetacea sp. are undefined species within the order Cetacea.

### Data validation and characterisation

We collated all known specimen and stranding records within Victorian waters up to 2016, however we found that records prior to 1920 lacked sufficient information to determine stranding status, location and/or date. We therefore excluded pre-1920 records from further analyses. In total, we sourced 10,116 occurrence records from Museums Victoria (n = 417), Zoos Victoria (n = 708), government and non-government online flora and fauna sighting/stranding databases (Victorian Biodiversity Atlas (VBA, n = 5,196), Atlas of Living Australia (ALA, n = 945), Australian Marine Mammal Centre (AMMC, n = 277), the Victorian Cetacean Stranding Network (VCSN, n = 98), the Marine Mammal Foundation (MMF, n = 231), Wildlife Victoria (n = 2,032) and the International Whaling Commission reports (n = 310).

As the stranding records included data recorded from a range of sources with non-consistent methodologies, we assigned a confidence rating of ‘low’, ‘medium’ and ‘high’ to each record based on species identification, date, and location accuracy, in accordance with Segawa and Kemper [14] and Meissner [35]. We removed all records which could not be confirmed as strandings, i.e. skeletal remains and potential sightings of free-swimming individuals. With respect to location data, all Victorian records were used for the species list, however, only records where the location of the stranding included GPS coordinates to 0.1 decimal degree or a description of the location which translates to equal or greater resolution (i.e. “Venus Bay, 400m north of Number 5 Beach Access”) were retained for statistical spatial analysis. When the precise date of the event was unspecified, the estimated month, based on record notes, was assigned. If this was not possible, we excluded the record from the seasonal analysis. We used only records with discernible year of stranding for the dataset if the record was in a similar geographical location to another record, to reduce the likelihood of duplicates.

Where available, information on age class and sex was also included. Age classes consisted of ‘immature’, ‘adult’, or ‘unknown’, and sex was classified as ‘male’, ‘female’, or ‘unknown’. Age class was assigned based on record notes, or morphological measurements within the record which allowed for classification. All stranding events were reported as ‘single’, ‘mother and calf’, or ‘mass’ (3 or more individuals [14, 15]) strandings. Conservation status was assigned to each species based on their IUCN Red List classification of ‘least concern’, ‘near threatened’, ‘vulnerable’, ‘endangered’, ‘critically endangered’, or ‘data deficient’. Given the newly described status and current paucity of long-term biological data on the species, *T*. *australis* is yet to be listed in the IUCN Red List. However given its listing as ‘Threatened’ in the Victorian Flora and Fauna Guarantee Act 1988, we classified the species as ‘Threatened’ for this study.

### Data analysis

Spatial and temporal patterns were investigated for pooled cetacean strandings and for the most common species across the stranding record. Decadal analysis was conducted using ANOVAs to compare decadal average annual stranding rates. Since the average annual stranding rate showed no difference after 1980, and was significantly higher than all prior decades, the temporal analysis was restricted to 1980–2016. Seasonal patterns were investigated for differences in austral seasons, defined in this study as southern hemisphere summer (December—February), autumn (March–May), winter (June–August) and spring (September–November).

For geographic analysis, stranding events were classified into mesoscale bioregions based on the geographic coordinates (OTW, CV, FLI, and TWO). The Gippsland Lakes (GL), a group of coastal lagoons within the TWO region contained a high number (n = 38) of strandings within its boundaries. This inshore waterway is not included in the IMCRA rationalisation, however it reflects differing environmental characteristics to the TWO oceanic coastline. We therefore treated it as a separate geographical region for the purposes of our spatial analysis. To account for any bias from regions with greater coastline lengths, the number of stranding events was divided by the length of coastline for each region to provide a stranding rate (number of stranding events per kilometre), as determined using QGIS 2.18.20 to interrogate the IMCRA v4 mesoscale bioregions and GL spatial layers.

To investigate spatial groupings along the open ocean coast, we compared the stranding rates between mesoscale bioregions which connected directly to the open ocean; OTW, CV, FLI, and TWO. To investigate if there were differences in species strandings between inshore and open ocean environments, we compared the stranding rate for VE and GL combined to pooled strandings from OTW, CV, FLI and TWO. The expected values used for statistical analysis were based on a standardised stranding rate, where the length of coastline in each region was multiplied by the average stranding rate across all coastal regions pooled.

We investigated differences in sex and age ratios where the unknown values for a given taxa accounted for less than 50% of the record.

### Statistical analysis

We used Chi-square and Fisher exact tests to test for significance in seasonal stranding patterns, spatial spread, age and sex class differences using R package ‘stats’ (version 3.5.0). Chi-square tests were used when 80% or more of the expected number of strandings for a given parameter was more than five. Where this assumption was not met, we used Fisher exact tests.

## Results

From 1920 to 2016, 424 verifiable cetacean stranding events containing 907 individuals were recorded within Victorian waters, across 31 cetacean species from seven families (Table 1). The most commonly reported stranded taxa were *Delphinus delphis* (n = 81), dolphins of the *Tursiops* genus (undefined *Tursiops* sp. n = 77; *T*. *australis*, n = 55; *T*. *truncatus*, n = 13; *Tursiops aduncus*, n = 1), *Physeter macrocephalus* (sperm whales, n = 34), *Globicephala* species (*Globicephala melas* (long-finned pilot whale), n = 12; *Globicephala sp*., n = 14), *Kogia breviceps* (pygmy sperm whale, n = 24), and *Megaptera novaeangliae* (humpback whale, n = 17) (Table 1). Five additional species have been added to previously reported species lists; *Tasmacetus sheperdi* (Shepherd's beaked whale), *Stenella coeruleoalba* (striped dolphin), *Steno bredanensis* (rough-toothed dolphin), *Kogia simus* (dwarf sperm whale), and the recently described *T*. *australis*. The conservation status of these 31 species included ‘least concern’ (n = 11), ‘endangered’ (n = 2), ‘threatened’ (n = 1), ‘vulnerable’ (n = 1), ‘near threatened’ (n = 1) and ‘data deficient’ (n = 15).

**Table 1:** Cetacean strandings in Victoria, Australia from 1920 to 2016.

Taxa	Common Name	Conservation status	Date Range	Events	Individuals	Stranding Type			Single Stranding Demographic					
						Single	M/C	Mass	Sex			Age Class		
									M	F	U	A	I	U
**Cetacea**														
Cetacea sp.	Undefined cetacean	N/A^a^	1961–1994	3	3	3	0	0	0	0	3	0	0	3
**Mysticete**														
**Balaenidae**														
*Eubalaena australis*	Southern right whale	LC	2013	1	1	1	0	0	0	0	1	0	1	0
**Balaenopteridae**														
*Balaenoptera* sp.	Rorqual whale	N/A^a^	1989	1	1	1	0	0	0	1	0	0	0	1
*Balaenoptera acutorostrata*	Common minke whale	LC	1946–2014	6	6	6	0	0	1	3	2	0	4	2
*Balaenoptera bonaerensis*	Antarctic minke whale	NT	1946	1	1	1	0		0	0	1			
*Balaenoptera edeni*	Bryde's whale	DD	1968–2000	4	4	4	0	0	1	1	2	1	2	1
*Balaenoptera musculus*	Blue whale	EN	1955–2014	8	8	8	0	0	4	1	3	1	4	3
*Balaenoptera physalus*	Fin whale	EN	1985–2014	4	4	4	0	0	0	1	3	0	1	3
*Megaptera novaeangliae*	Humpback whale	LC	1978–2014	17	17	17	0	0	4	2	11	4	3	10
**Neobalaenidae**														
*Caperea marginata*	Pygmy right whale	DD	1946–2015	8	8	8	0	0	4	1	3	1	6	1
**Odontocete**														
**Delphinidae**														
*Delphinid sp*.	Undefined dolphin	N/A^a^	1984–2016	4	4	4	0	0	1	0	3	0	0	4
*Delphinus delphis*	Short-beaked common dolphin	LC	1959–2016	81	81	81	0	0	15	22	44	24	19	38
*Globicephala sp*.	Short-finned or long-finned pilot whale	N/A^a^	1946–2004	14	342	12	0	2	0	0	12	1	0	11
*Globicephala melas*	Long-finned pilot whale	DD	1964–2004	12	31	11	0	3	6	1	4	3	5	3
*Grampus griseus*	Risso's dolphin	LC	1926–2014	5	5	5	0	0	2	0	3	4	0	1
*Lagenodelphis hosei*	Fraser's dolphin	LC	1978	1	3	0	0	1	0	0	0	0	0	0
*Orcinus orca*	Killer whale	DD	1933–2013	8	8	8	0	0	3	2	3	2	2	4
*Pseudorca crassidens*	False killer whale	DD	1965–2011	3	89	2	0	1	0	1	1	0	0	2
*Stenella coeruleoalba*	Striped dolphin	LC	2011–2014	3	3	3	0	0	0	2	1	1	1	1
*Steno bredanensis*	Rough-toothed dolphin	LC	2012	1	1	1	0	0	1	0	0	0	1	0
*Tursiops* sp.	Undefined bottlenose dolphin or Burrunan dolphin	N/A^a^	1932–2014	77	80	74	3	0	8	7	59	12	6	56
*Tursiops aduncus*	Indo-Pacific bottlenose dolphin	DD	2008	1	1	1	0	0	0	1	0	0	0	1
*Tursiops australis*	Burrunan dolphin	T	1967–2015	55	56	54	1	0	33	13	8	33	11	10
*Tursiops truncatus*	Common bottlenose dolphin	LC	1988–2014	13	17	12	0	1	3	8	1	10	1	1
**Kogiidae**														
*Kogia* sp.	Pygmy sperm whale or dwarf sperm whale	N/A^a^	1954–2004	4	4	4	0	0	0	0	4	0	0	4
*Kogia breviceps*	Pygmy sperm whale	DD	1975–2016	19	19	19	0	0	7	5	7	7	3	9
*Kogia simus*	Dwarf sperm whale	DD	2016	1	1	1	0	0	0	0	1	1	0	0
**Physeteridae**														
*Physeter macrocephalus*	Sperm whale	VU	1920–2009	34	70	33	0	1	5	3	25	4	3	26
**Ziphiidae**														
*Ziphiidae sp*.	Beaked whales	N/A^a^	1986–1988	3	3	3	0	0	0	0	3	0	0	3
*Hyperoodon planifrons*	Southern bottlenose whale	LC	1950–1992	2	2	2	0	0	1	0	1	0	1	1
*Mesoplodon bowdoini*	Andrew's beaked whale	DD	1968	1	1	1	0	0	0	1	0	1	0	0
*Mesoplodon densirostris*	Blainville's beaked whale	DD	1990–2008	2	2	2	0	0	0	2	0	1	1	0
*Mesoplodon ginkgodens*	Ginkgo-toothed beaked whale	DD	1983	1	1	1	0	0	0	0	1	0	0	1
*Mesoplodon grayi*	Gray's beaked whale	DD	1982–2010	6	8	4	2	0	1	2	1	1	2	1
*Mesoplodon layardii*	Strap-toothed whale	DD	1955–2004	12	12	12	0	0	1	6	5	7	1	4
*Mesoplodon mirus*	True's beaked whale	DD	1980	1	1	1	0	0	0	1	0	1	0	0
*Tasmacetus shepherdi*	Shepherd's beaked whale	DD	2012–2014	2	2	2	0	0	0	1	1	1	1	0
*Ziphius cavirostris*	Cuvier's beaked whale	LC	1964–2008	5	5	5	0	0	1	2	2	2	2	1
***Total***			**1920–2016**	**424**	**905**	**411**	**6**	**7**	**102**	**90**	**219**	**123**	**82**	**206**

M/C, mother and calf stranding; M, male; F, female; A, Adult; I, Immature; U, unknown;

^a^ Data not available as the taxa includes multiple species with the potential to have varying conservation status’.

Of the 424 stranding events, the majority (411) were recorded as single strandings; seven mass strandings were recorded and six were mother and calf strandings (Table 1). The seven mass stranding events were all single species events; *G*. *melas* (single event, n(individuals) = 20), *Globicephala sp*. (two events, n(individuals) = 140, n(individuals) = 190), *Lagenodelphis hosei* (Fraser’s dolphin, single event, n(individuals) = 3), *P*. *macrocephalus* (single event, n(individuals) = 37), *Pseudorca crassidens* (false killer whale, single event, n(individuals) = 87) and *T*. *truncatus* (single event, n(individuals) = 5) (Table 1). The six mother and calf strandings were recorded across three taxa; *Mesoplodon grayi* (Gray’s beaked whale, n = 2), *T*. *australis* (n = 1) and undefined *Tursiops* sp. (n = 3).

### Age and sex class

Of the 411 single stranding records, 178 (43%) did not have any age or sex class assigned, or a quantitative measure that could be used to infer demographic class. Although 233 (57%) records had some information, only 164 (40%) had both age and sex class recorded. In strandings where age was recorded (n = 205), adults (n = 123) stranded more often than immature individuals (n = 82; P < 0.01, X^2^ = 8.20). Of the taxa analysed, *T*. *australis* (P < 0.01, X^2^ = 11) and *T*. *truncatus* (P < 0.01, X^2^ = 7.4) were the only two to demonstrate a significant age ratio; both had a larger proportion of adults strand than immature individuals (Table 1). When differences within sex ratios were tested only *T*. *australis* demonstrated a significant difference (P < 0.01, X^2^ = 8.7), with more males (n = 33) than females (n = 13).

Interestingly, whilst not tested due to sample size, *Mesoplodon layardii* (strap-toothed whales) individuals were almost exclusively female (6:1) and had a 7:1 ratio of adult to immature individuals which stranded (Table 1).

### Temporal patterns

**Fig 2:**
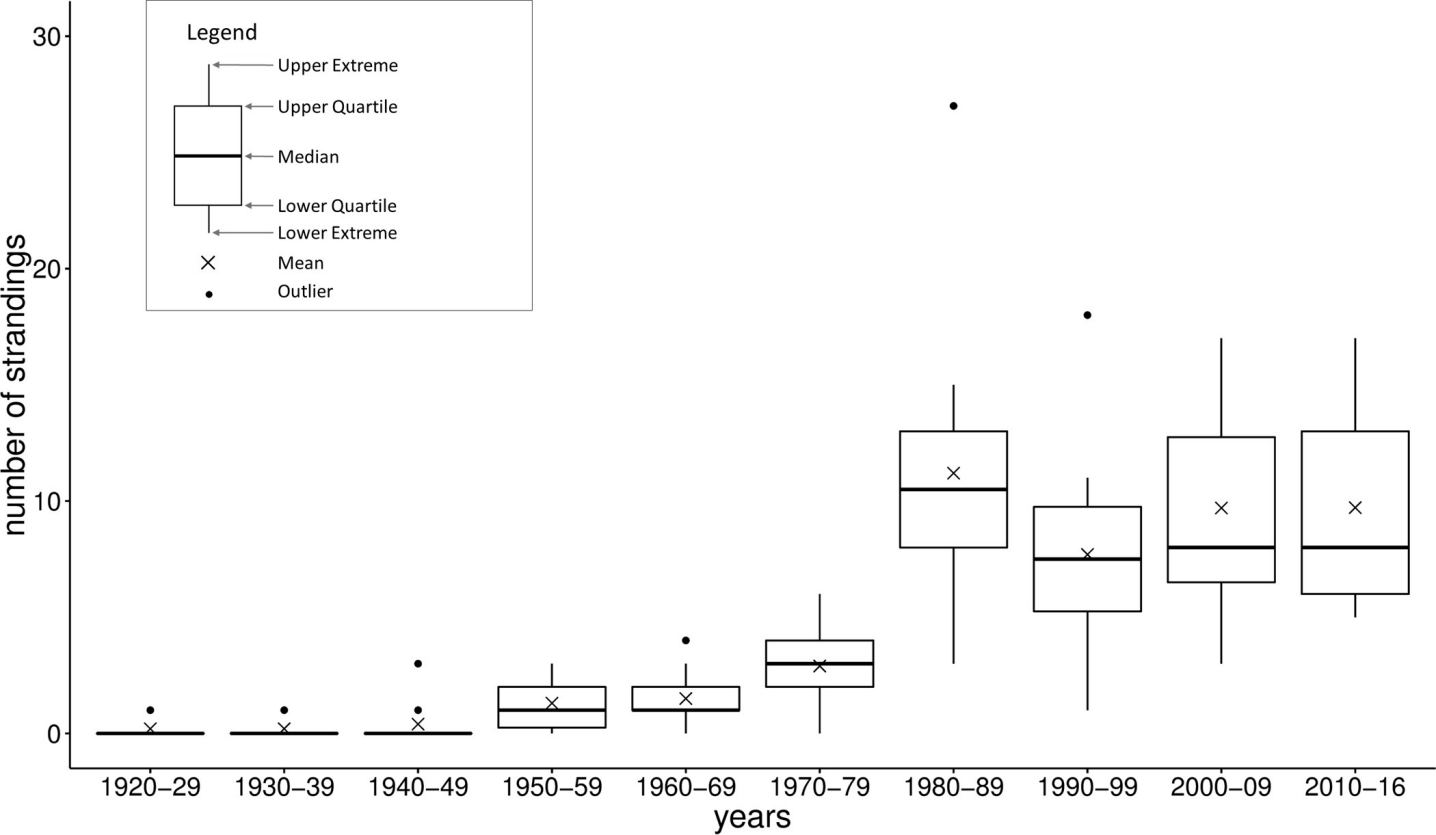
Decadal cetacean stranding records, reported in Victoria, Australia (1920–2016) (n = 423). Results for 2010–2016 were calculated from seven years of data.

Victoria’s stranding record spans from 1920 to 2016 with strandings recorded every year from 1974 to 2016 (S1 Fig). The average number of stranding events per year was 4.3 (SD ± 5.4), with the highest number recorded in 1988 (n = 27). The average annual number of stranding events per decade more than tripled from the 1970s (2.8 per year) to the 1980s (11.2 per year; Fig 2). The average annual stranding rate per decade was consistent across the four latter time periods; 1980–89, 1990–99, 2000–09 and 2010–16 (F (3,33) = 0.712, P = 0.552).

**Fig 3:**
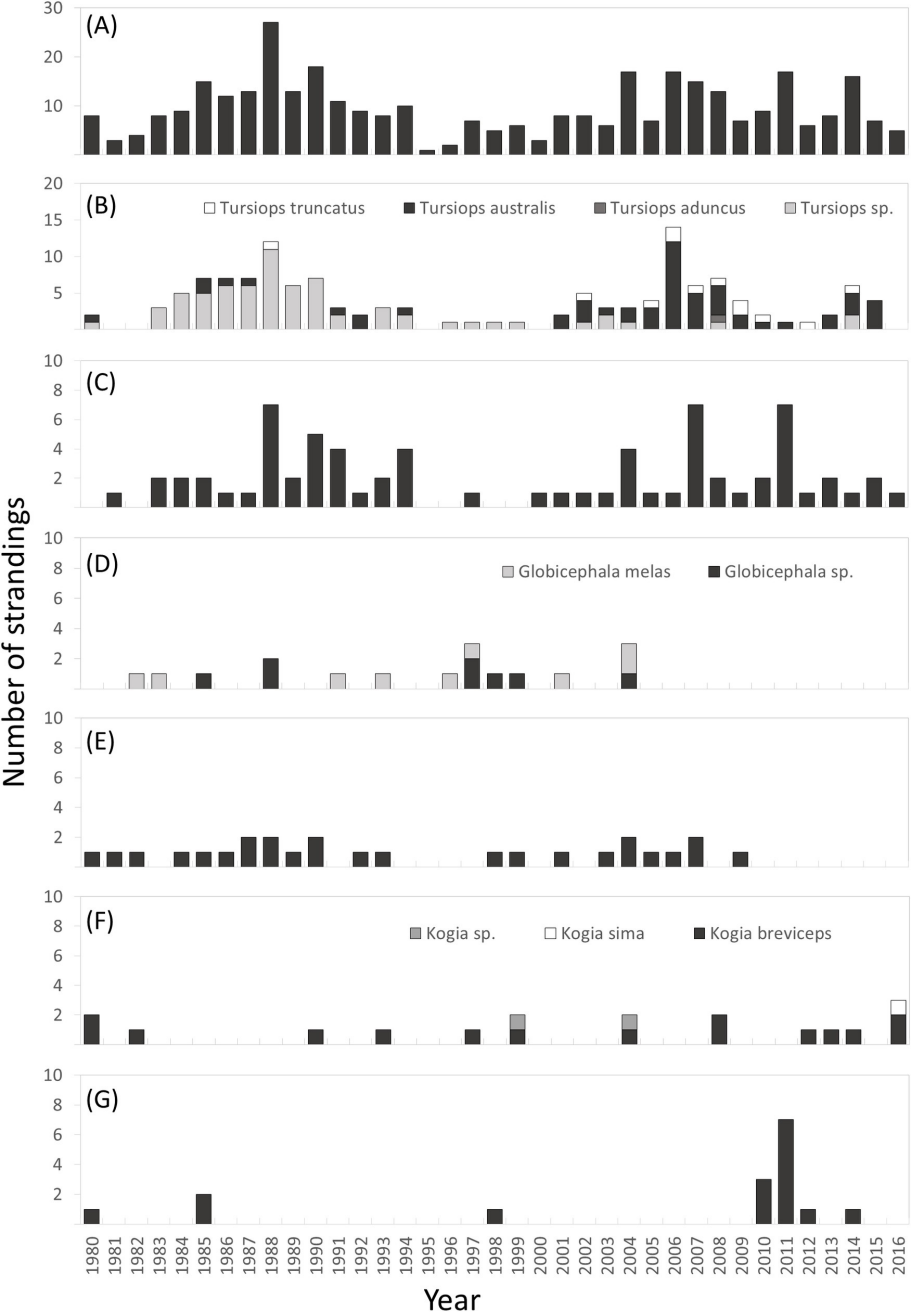
Annual strandings for all cetacea and commonly reported taxa, Victoria, Australia (1980–2016). (A) Cetacea (n = 358), (B) *Tursiops* species (n = 135), (C) *Delphinus delphis* (n = 71), (D) *Globicephala species* (n = 17), (E) *Physeter macrocephalus* (n = 26), (F) *Kogia species* (n = 18), (G) *Megaptera novaeangliae* (n = 15).

The most commonly recorded taxa demonstrated differing interannual stranding patterns (Fig 3). *Tursiops spp*. and *D*. *delphis* had two peaks in annual stranding rates; one in the 1980’s and one in the 2000’s. Prior to 2001 the *Tursiops spp*. record was dominated by unidentified *Tursiops* species, however post 2001 the record was dominated by *T*. *australis* (Fig 3). *Megaptera noveangliae* had a peak in 2011, and no *Globicephala spp*. strandings were recorded after 2004 (Fig 3). *Kogia spp*. stranded most frequently in 2016 with both *K*. *breviceps* and *K*. *sima* recorded. Finally, *P*. *macrocephalus* stranded with some consistency at low frequency until 2009, with no strandings recorded after this date.

**Fig 4:**
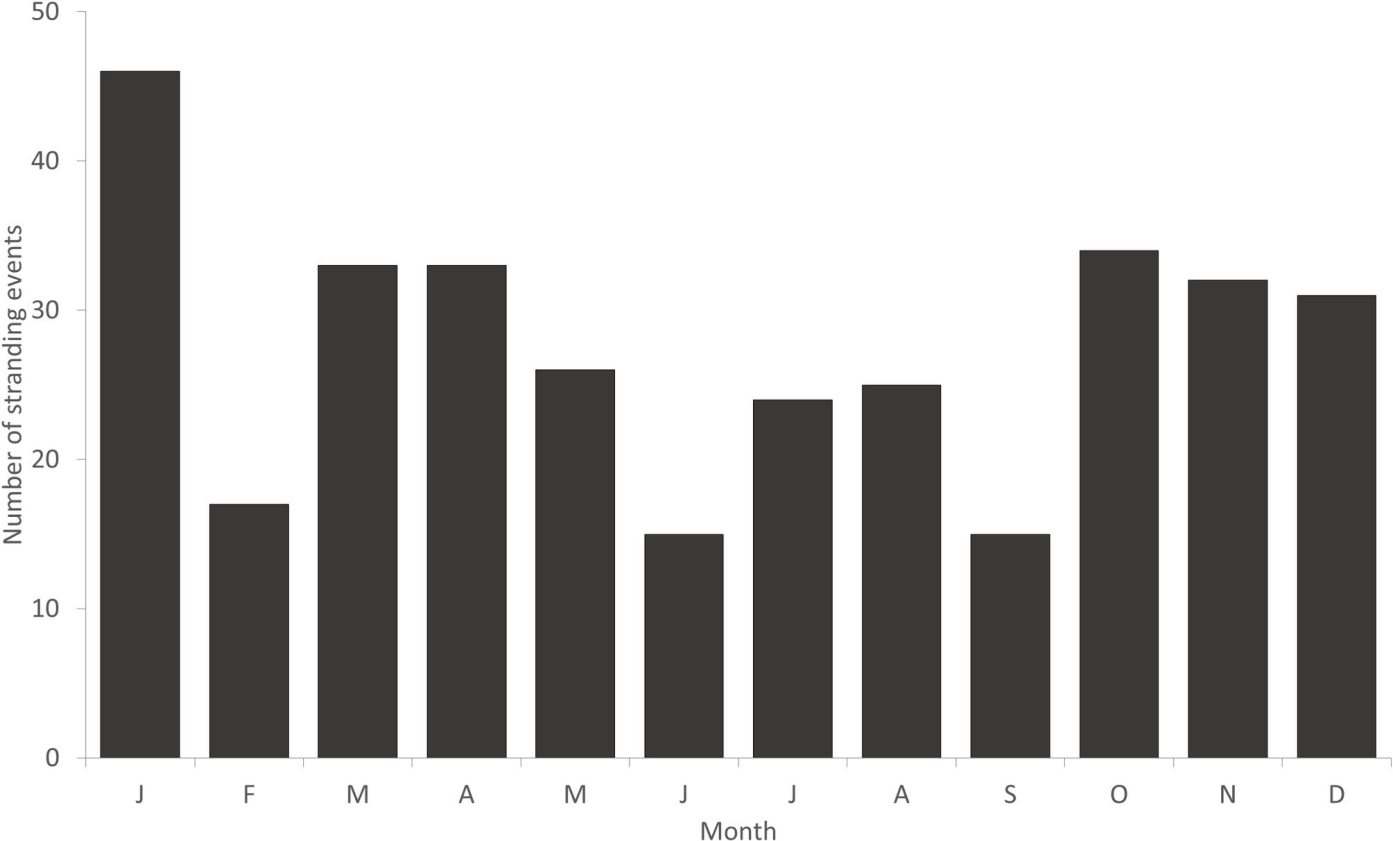
Monthly cetacean stranding events reported in Victoria, Australia (1980–2016), (n = 331).

**Fig 5:**
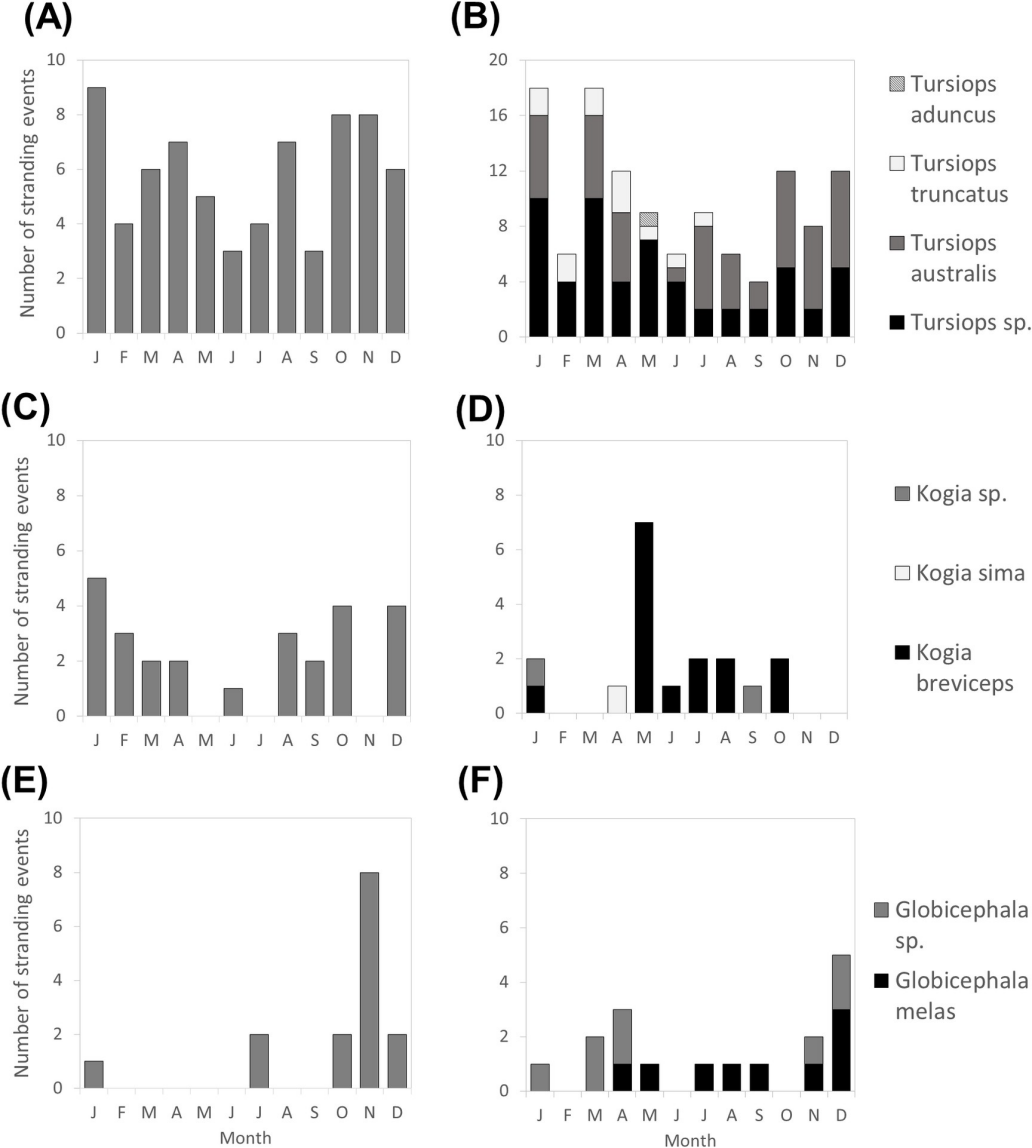
Monthly stranding events reported in Victoria, Australia (1980–2016). (A) *Delphinus delphis* (n = 70), (B) *Tursiops species* (n = 120), (C) *Physeter macrocephalus* (n = 26), (D) *Kogia species* (n = 18), (E) *Megaptera novaeangliae* (n = 15), (F) *Globicephala species* (n = 17).

Cetacean strandings were reported with relative equality across all seasons between 1980 and 2016 (n = 331, P = 0.077, X^2^ = 6.85, Fig 4). We found no significant seasonal pattern for any taxa tested (Fig 5). Whilst not significant, there was a high occurrence of *M*. *noveangliae* in November with eight of the 15 strandings occurring in this month. Additionally, *K*. *breviceps* demonstrated a peak (39%) in strandings during May with 7 of the 15 strandings occurring in this month (Fig 5).

### Spatial patterns

**Fig 6:**
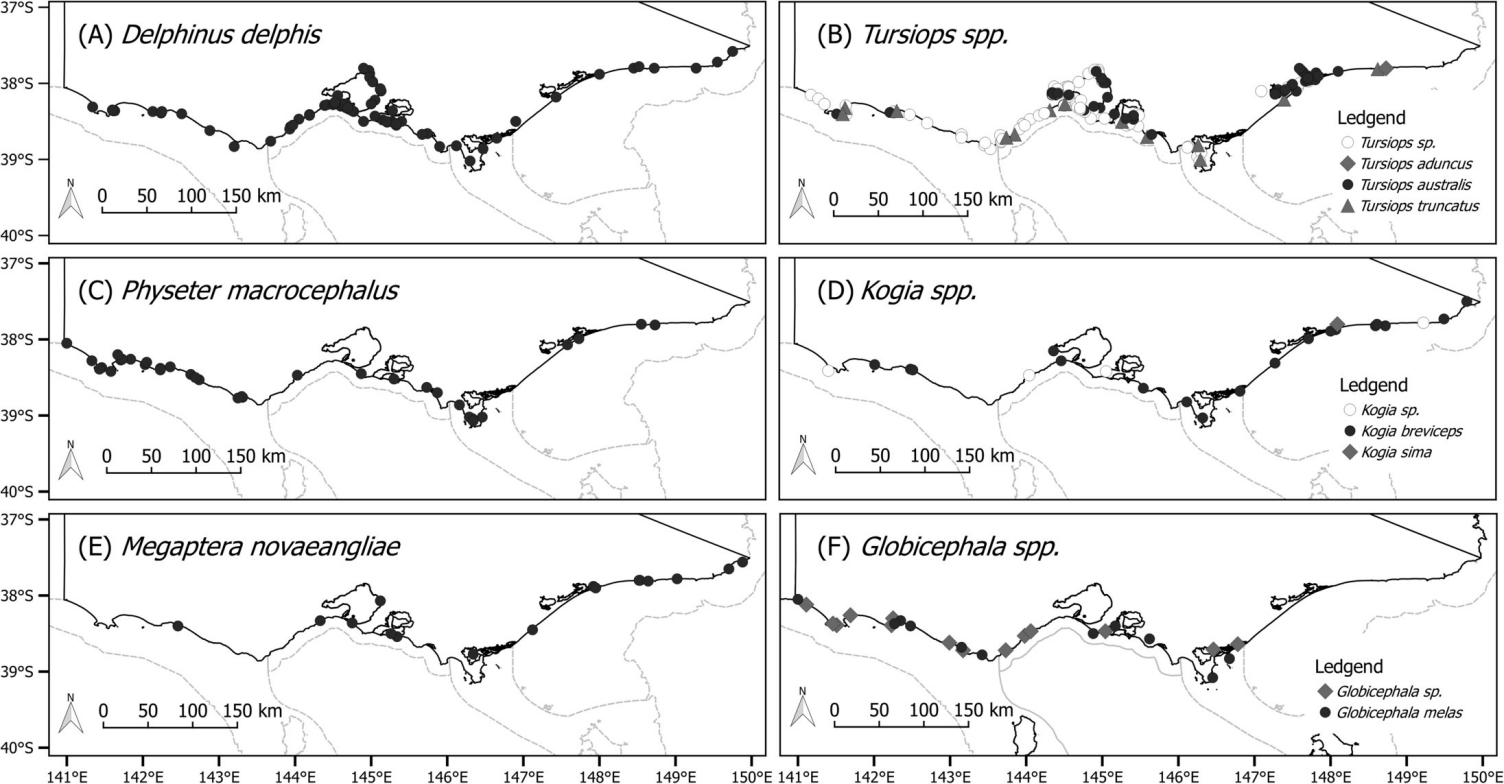
**Stranding events of commonly recorded taxa, Victoria, Australia (1920–2016).** (A) *Delphinus delphis* (n = 81), (B) *Tursiops* species (n = 146), (C) *Physeter macrocephalus* (n = 26), (D) *Kogia species* (n = 18), (E) *Megaptera novaeangliae* (n = 16), (F) *Globicephala species* (n = 26). Reproduced from Commonwealth of Australia, Australian Government Department of Environment 2016 licensed for re-use under CC BY 4.0., and Victorian State Government, Department of Environment, Land, Water and Planning, licensed for re-use under CC BY 4.0.

The majority of strandings and the greatest species richness occurred within the Otways (OTW) region with 105 (0.24 strandings/km) of 284 open ocean stranding events, and 24 of the total 31 species recorded. Stranding events were not evenly distributed along the open ocean mesoscale bioregions (P < 0.01, X^2^ = 28.86). We found a significant spatial pattern along the open ocean coast for D. *delphis* (P < 0.01, X^2^ = 27.70) with the highest incidence of stranding events in the Central Victoria (CV) region (n = 34, 0.08 strandings per km, Fig 6). Stranding events were greatest for *P*. *macrocephalus* and *Globicephala spp*. in the OTW region (Fig 6), 20 of the 34 *P*. *macrocephalus* strandings (0.05 strandings per km, P < 0.05), 15 of the 26 *Globicephala spp*. strandings (0.04 strandings per km, P < 0.05) were recorded. For the remaining taxa, we found no significant spatial pattern of strandings; however, nine of the 17 stranding events for *M*. *noveangliae* occurred in the TWO region (Fig 6). Of note, the three *Globicephala spp*. mass strandings were all within a 35km radius of one another in FLI (S2 Fig).

Stranding rates between those of the pooled embayments and inshore regions of VE and GL and the pooled open ocean regions (OTW, CV, FLI, and TWO) differed for all tested taxa (Fig 6). *T*. *australis* however, was the only species to demonstrate a higher stranding rate in the VE and GL region than that of the open ocean coast (Fig 6).

We found that three species were recorded outside their previously-documented range [36]; *Steno bredanensis* (rough-toothed dolphin, n = 1), *Stenella coeruleoalba* (striped dolphin, n = 3), and *L*. *hosei* (n = 1). A single stranding of a *S*. *bredanensis* in 2012 provided the only recorded presence of the species in Victoria, with the exception of a skeletal museum specimen from the 1800s (not included in the record due to confidence ratings). The *L*. *hosei* stranding was classified as a mass stranding, consisting of three adult individuals.

## Discussion

The use of stranding information can provide valuable data on cetacean species diversity within a region [3]. Here we provide the first description of spatial and temporal stranding records for all Cetacea in Victoria from 1920–2016. Our results confirm the presence of 31 species, building on previously reported species counts of 26 [14, 37] and 23 [17] and extending this list to include five additional species not previously reported. With almost half (48%) of the species stranded being classified as data deficient, this record greatly expands the knowledge for the region on the occurrence of cetaceans in Victoria and highlights important demographic and temporal patterns in their distribution.

The diversity observed in Victoria is high when compared with international stranding records [38] but is consistent with records for neighbouring states and within the South East Australian region; South Australia, 31 species [14]; Tasmania, 28 species [37]; New South Wales, 33 species [16]. Whilst overall diversity is high, the actual number of stranding events is relatively low when compared with other Australian stranding records [4, 14–16] and those in North America [7, 38, 39].

An increase in stranding records with time has been observed in a number of studies and is generally attributed to an increase in observer effort [4, 6, 40]. It is likely that the increase in stranding rate from the 1970s to the 1980s in this study is reflective of a similar increase in reporting effort. However, whilst neighbouring states, South Australia [14] and New South Wales [16], have observed an upwards trend in average annual stranding events per decade, in Victoria this measure is consistent from the 1980s through to the 2010s, with high interannual variability. The interannual variability is driven largely by the commonly stranded dolphin taxa, which had a high number of stranding events in the 1980s, and 2000s with comparatively low reports through the 1990s. Evans and Thresher [17] reported a similar pattern for all cetacean strandings in the state prior to 1980, finding a periodic pattern of strandings at an 11.2 year interval, attributing this to changes in sea surface pressure. To gain a deeper understanding of the potential drivers behind the interannual patterns observed, further research investigating the environmental drivers of distribution such as sea surface temperature, sea surface pressure, eddy kinetic energy and *chlorophyll-a* would be beneficial.

Disproportional observational effort has been thought to drive temporal patterns and differences in spatial variation in stranding density [7, 16]. Within Victoria the human population is centred around Melbourne and Geelong [41], both of which are adjacent to Port Phillip Bay, a large embayment part of the VE region. However, *T*. *australis* was the only taxa to have a higher stranding rate along these highly populated coastlines within the inshore and embayment areas of VE and GL when compared to other regions. This species has known resident populations within Port Phillip Bay and GL with high site fidelity [28, 42, 43], and therefore it is not unexpected to see a higher stranding rate within these areas.

Other than observational effort, the oceanographic characteristics of an area are often attributed to the differences observed in cetacean distribution and diversity [44, 45]. In this study the OTW region demonstrated the highest diversity and the highest stranding rate of cetaceans along the open ocean coastal regions. This region contains the Bonney Upwelling, one of the most intense and productive regions within Australia [46], and has a narrow continental shelf with a steep offshore gradient [47]. Previous studies have reported high cetacean diversity correlated with upwellings due to high levels of associated prey [45, 48] and the presence of deep water close to shore providing a near shore habitat for pelagic and deep diving species [49]. Both *Globicephala spp*. and *P*. *macrocephalus* are examples of such pelagic and deep diving species and had the highest stranding incidence in the region, which occurred during the upwelling seasons. Both species’ distribution having been correlated to prey related drivers in previous studies in the Sargasso Sea and north-east Atlantic Ocean [50, 51]

For data deficient species, such as most beaked whales, strandings provide the primary data source for information [4, 16]. Beaked whales contributed over 7% of stranding events in Victoria across nine species, however many of these species have never been sighted alive within state waters and are difficult to identify at sea [52]. Collecting demographic information is particularly important for these species in which the life cycle and distribution is not accurately known, as it can help identify areas of significance and habitat use [53, 54]. *Mesoplodon layardii* and *M*. *grayi* are the most commonly stranded beaked whales in Australia [55]; we found that this is also reflected in the current Victorian record. For *M*. *layardii* females and adults were the most common, and the *M*. *grayi* record had two mother and calf strandings of the six events recorded for the species. Whilst there were still a number of unknown age and sex class individuals from these taxa, this information provides valuable insights to the potential demographics of the wild population of these species with the region.

Previous studies have found a higher stranding rate for immature cetaceans than adults, hypothesised to be linked to natural attrition rates and varying habitat use during early life stages, [15, 38, 56]. The overall Victorian cetacean stranding record demonstrated the opposite; we found a significantly greater number of adults stranding than that of immature individuals. However, the high numbers of *T*. *australis* and *D*. *delphis* strandings drove this pattern, with no other taxa demonstrating a significant age ratio. *T*. *australis* was the only taxa to demonstrate a significant sex ratio, with a predominance of males. It is worth noting that *T*. *australis* was one of the only taxa which had a relatively high proportion of strandings with age or sex class information; the lack of significant results for other taxa may be a reflection on the paucity of data. Demographic information is required across a higher proportion of stranding events in order to accurately infer ecologically significant trends for the cetaceans within this region.

Stranding data often reflects known migration pathways of cetaceans. *Megaptera novaeangliae* are known to migrate through Victoria northward from April to August from their Antarctic feeding to tropical breeding grounds, and travelling southward between October and December [19, 57]. Whilst a high number of sightings are recorded for the taxa on their northerly migration [58] the majority of strandings occurred in November, coinciding with the populations’ southward migration. Reduced fitness due to nutritional stress could explain this pattern with previous work suggesting that both female and juvenile individuals are more likely to strand on the way from calving to feeding grounds (southward migration) as females have often depleted their energy stores [10]. Further, the incidence of stranding was high in 2011, coinciding with a significant peak in persistent organic pollutant contaminant burdens and lowered observed body condition for the cohort [59]. Determining the cause of these strandings is important to make inferences of the wild population, and therefore collecting age, sex, body condition and cause of death at each stranding event is crucial.

Whilst other regions have been able to make inferences on population demographics and habitat use from stranding data, the paucity of information collected and stored for each stranding event in this record is such that for most taxa this is not feasible. For example, *K*. *breviceps* in New Zealand are the most commonly stranded cetacean taxa and are dominated by females and mother and calf strandings. This observation has led researchers to suggest there is a breeding ground nearby [60]. In Victoria, *K*. *breviceps* were one of the most commonly recorded species, however in 47% (n = 9) of these strandings, age was unknown, and in 37% (n = 7) of events sex class was undetermined. While our study provides vital information on where and when *K*. *breviceps* may be in higher abundances, given the lack of demographic information in this record it is unclear as to what sort of population exists in Victoria.

As we have demonstrated, stranding records are useful for giving an indication of species presence within a region, can be used to identify patterns in diversity and distribution of cetaceans, and importantly provide valuable information related to data deficient species, such as beaked whales and *K*. *breviceps*. This record also forms baseline information by which we can monitor change in assemblages and distribution across the region, as has been documented with global climate change in other regions [61]. Finally, the information collected from strandings of commonly stranded dolphin species, such as *T*. *australis*, is of value not only to increase the understanding of cetacean communities, but also to provide information on attrition rates as part of population viability analysis required for species conservation status classifications.

We acknowledge there are innate limitations and biases in stranding data which may impact on the inferences made. The likelihood of an individual stranding is dependent on; the habitat, with inshore species more likely to be detected as offshore mortalities are often predated, sink or decompose prior to stranding [39]; current and wave action affect the likelihood of a carcass washing ashore [3]; and observational biases, with coastlines which have high human use more likely to have high detectability, compared with remote coastlines [16]. Further, the absence of consistently-collected descriptive data (e.g., confirmed species identification via morphological specimens and/or genetics, sex and age class) recorded from each stranding event limits the inference that can be made from these data. With only 40% of the Victorian stranding record containing both sex and age class information, and 106 events comprised of 439 individuals pertaining to records with undefined species data due to lack of morphological, genetic or skeletal information, we can make limited generalisations about population dynamics, status, and life history of these species.

### Stranding data guidelines

Based on the outcomes of this study, we recommend standardised protocols for the collection of data and biological samples for each stranding event, as exampled within Australian government documentation [62]. This includes the collection of consistent and useful morphological measurements, sex, age class, body health condition, decomposition state, evidence of human interaction, samples for toxicological and genetic analysis, photographs and GPS coordinates, alongside full necropsies including pathology. Understanding that this may not always be feasible or practicable, at a minimum we recommend, total length, sex, age class, body health condition, decomposition state, a skin sample, GPS coordinates and photographs to be collected. All physical sampling should only be conducted by those with appropriate experience, qualification and permits to do so. In line with Bates and Pecl [63], we recommend any stranding data be validated by reputable marine mammologists capable of species identification. Finally, we recommend depositing stranding data in an actively managed, user-friendly and accessible database, which is regularly updated and vetted by a governing body and specimens deposited in an actively maintained and accessible museum collection. Collaborations between the various marine mammal researchers and governing bodies are also encouraged, with regular workshops allowing current findings and advances in knowledge to be more easily disseminated and effectively communicated.

## Supporting information

S1 Fig Annual cetacean strandings recorded in Victoria, Australia (1920–2016). (TIF)

S2 Fig Cetacean mass stranding events recorded in Victoria, Australia (1920–2016). (TIF)

S1 File Cetacean stranding records for Victoria, Australia. (XLSX)
