# Supplementary figures and images for: Cetacean biodiversity, spatial and temporal trends based on stranding records (1920-2016), Victoria, Australia
Source: PLoS One. 2019 Oct 10;14(10):e0223712. doi: 10.1371/journal.pone.0223712 (PMC6786658; doi:10.1371/journal.pone.0223712)

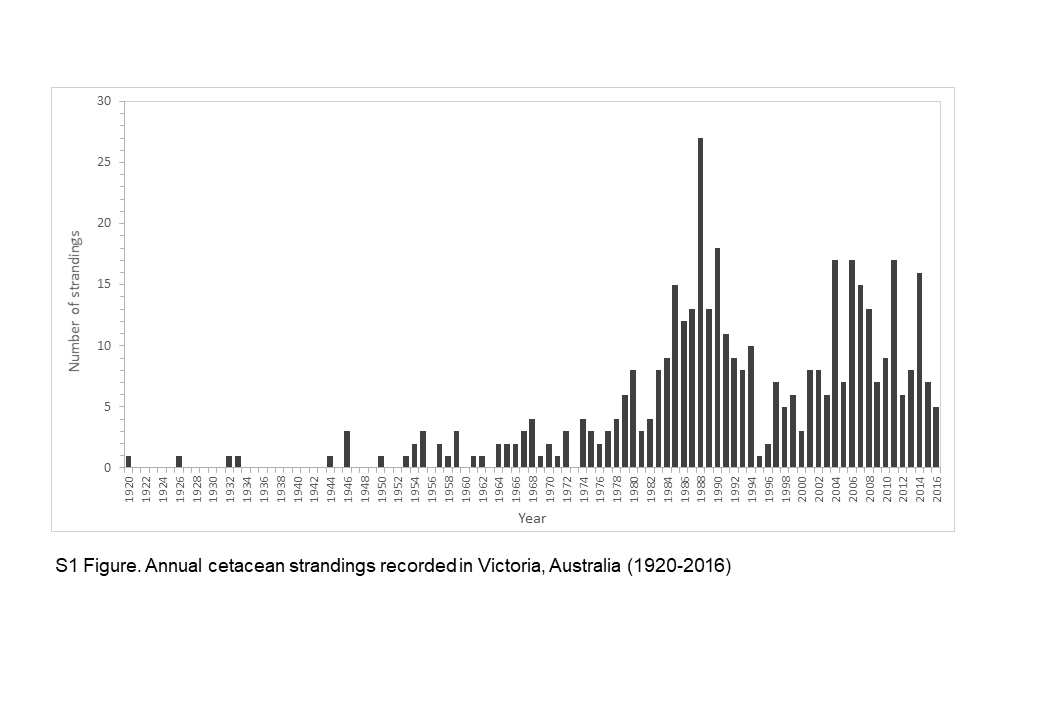

Supplement: S1 Fig — (TIF) [file pone.0223712.s001.TIF]

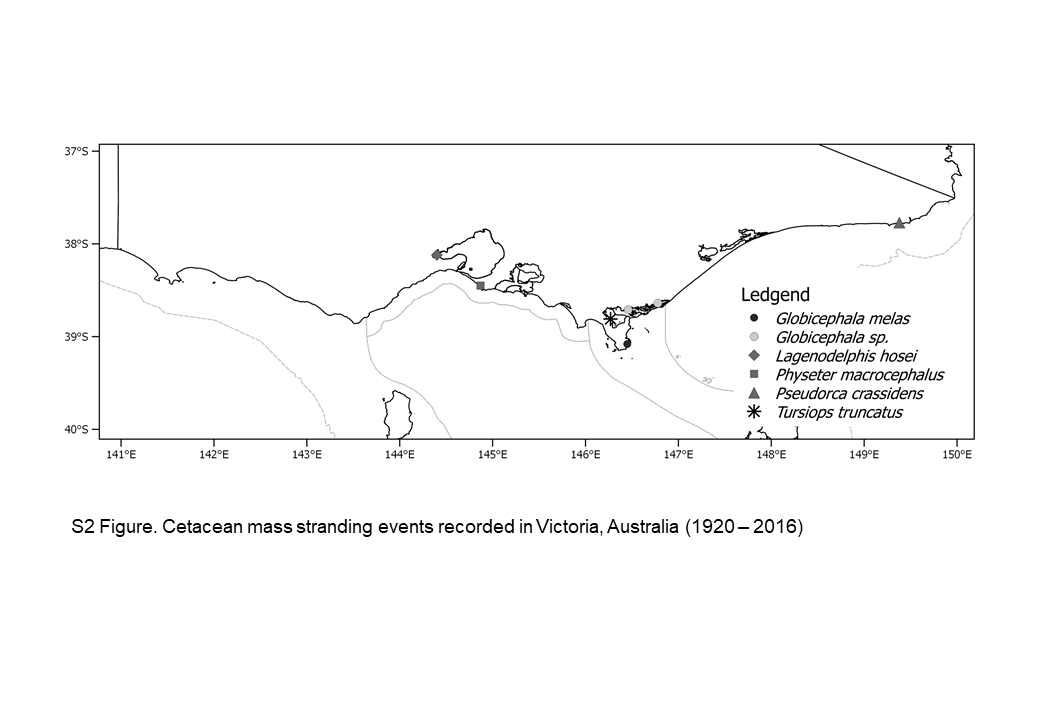

Supplement: S2 Fig — (TIF) [file pone.0223712.s002.TIF]
